# Real world community-based HIV Rapid Start Antiretroviral with B/F/TAF versus prior models of antiretroviral therapy start – the RoCHaCHa study, a pilot study

**DOI:** 10.1186/s12981-024-00631-6

**Published:** 2024-07-10

**Authors:** William Valenti, Jacob Scutaru, Michael Mancenido, Ashley Zuppelli, Alexandra Danforth, Roberto Corales, Shealynn Hilliard

**Affiliations:** 1 Trillium Health, 259 Monroe Ave Suite 100, Rochester, NY 14607 USA; 2 Gilead Sciences, 333 Lakeside Drive, Foster City, CA 94404 USA

**Keywords:** HIV, ART, Rapid start ART, Community-based health care, HIV viral load suppression

## Abstract

**Background:**

The rapid start of antiretroviral therapy (RSA) model initiates antiretroviral therapy (ART) as soon as possible after a new or preliminary diagnosis of HIV, in advance of HIV-1 RNA and other baseline laboratory testing. This observational study aims to determine if RSA with a single tablet regimen of bictegravir, emtricitabine, and tenofovir alafenamide (B/F/TAF) is an effective regimen for achieving viral suppression and accepted by patients at the time of diagnosis.

**Methods:**

Adults newly or preliminarily diagnosed with HIV were enrolled from October 2018 through September 2021. Real world advantage, measured in days between clinical milestones and time to virologic suppression, associated with B/F/TAF RSA was compared to historical controls.

**Results:**

All Study RSA participants (*n* = 45) accepted treatment at their first visit and 43(95.6%) achieved virologic suppression by week 48. Study RSA participants had a significantly shorter time (median 32 days) from diagnosis to ART initiation and virologic suppression, in comparison to historical controls (median 181 days) (*n* = 42). Qualitative feedback from study RSA participants showed high acceptance positive response to RSA.

**Conclusions:**

RSA is feasible and well accepted by patients in a real-world community-based clinic setting. Promoting RSA in community-based clinics is an important tool in ending the HIV epidemic.

**Supplementary Information:**

The online version contains supplementary material available at 10.1186/s12981-024-00631-6.

## Background

Trillium Health (Trillium) is a community health center in Rochester, NY with a more than 30-year legacy of HIV prevention and treatment. Trillium serves about 850 people living with HIV and maintains several robust programs as part of New York State’s initiative to End the HIV Epidemic [1] and the national initiative, Ending the HIV Epidemic in the U.S. [2], which highlight the importance of engaging and retaining people living with HIV (PLWH) in care. In January of 2017, Trillium began rapid start of antiretroviral therapy (RSA) for HIV treatment as standard of care. This treatment model initiates antiretroviral therapy (ART) the same day or as soon as possible after either a reactive rapid point-of-care HIV test or confirmatory HIV antibody test, in advance of HIV-1 RNA and other baseline laboratory testing. RSA truncates the time from HIV diagnosis to ART initiation. Research has shown that RSA leads to improved clinical outcomes, including higher retention in care and viral suppression, as compared to prior models of ART initiation [3–12].

On February 8, 2018, a single tablet regimen of bictegravir, emtricitabine, and tenofovir alafenamide (B/F/TAF) was approved by the U.S. Food and Drug Administration (FDA) for treatment of HIV-1 infection [13]. B/F/TAF is well-suited for RSA as it is one of few regimens that can be initiated without baseline laboratory results, as supported by efficacy and safety data from pre- and post-marketing trials [14–16]. The RoCHaCHa study bolsters the current literature through real-world data quantifying the difference in patient outcomes as compared to older models of care, as well as qualitative data on patient experience and acceptance of RSA.

This observational study’s objectives are to (1) determine if RSA with a single tablet regimen of B/F/TAF is an effective regimen for suppressing HIV-1 RNA to undetectable levels, and (2) assess patient acceptance of RSA at the time of diagnosis.

Real world advantage, measured in days between clinical milestones and time to virologic suppression, associated with B/F/TAF RSA was compared to historical controls. In addition, this study sought to identify areas of opportunity in the RSA workflow to shorten time to ART initiation and increase patient satisfaction with the RSA process.

## Methods

All individuals who presented to Trillium with a newly confirmed or preliminary HIV diagnosis from October 2018 through September 2021 were screened for this study. Participants were enrolled consecutively and had to meet the inclusion criteria of being at least 18 years of age, treatment naïve, and presenting to Trillium for treatment within 21 days of receiving their HIV diagnosis. Confirmatory laboratory results and resistance testing were not required for RSA initiation. Exclusion criteria included individuals who were less than 18 years old, had a previous HIV diagnosis and/or history of ART, declined RSA, were unable or unwilling to consent to study participation, could not present in person for study consent (i.e. in cases of incarceration or use of telemedicine), were pregnant or breastfeeding at presentation, were seen by a provider who was not trained on the study protocol, or were deemed medically inappropriate for B/F/TAF based on prescribing guidelines or the presence of medical conditions that the investigator believed would interfere with their ability to participate in the protocol. Persons who were not eligible for study participation were still offered RSA. Participants underwent baseline assessment and began a single tablet regimen of B/F/TAF on the day of clinic presentation. Trillium’s RSA process includes treatment adherence counseling, health insurance evaluation/navigation, management of social determinants of health, and onsite pharmacy and clinical laboratory services. Follow-up visits were conducted per protocol through 48 weeks at Trillium’s main clinic location in downtown Rochester, NY or via telemedicine.

The primary study endpoint is the time to virologic suppression. Secondary endpoints include proportion of patients who reached HIV-1 viral suppression to < 50 copies/mL within 48 weeks of ART initiation and quantified real-world treatment advantage, measure as time from diagnosis to clinic presentation and time from diagnosis to start of ART. Time to virologic suppression was measured as median days from ART initiation to a viral load of < 200 copies/mL and < 50 copies/mL. The viral suppression endpoint of < 200 copies/mL is standard in the literature to represent untransmissible/untransmittable status [17], and < 50 copies/mL is the typical clinical standard for virologic suppression [18, 19].

This study also identified barriers to initiation and adherence to ART, and participant acceptance of RSA. Participant feedback was collected through a questionnaire developed by the authors and administered at study completion, which asked about perceived barriers to medication initiation, attitudes about starting medication on the day of diagnosis or preliminary diagnosis, and potential interventions to improve the RSA process.

The historical control group comprised all the adult patients who presented to Trillium with a new HIV diagnosis and received standard of care ART initiation between January 2014 and August 2017, prior to RSA implementation at Trillium. The historical standard of care did not specify how quickly someone should be engaged in care and started on ART. Accordingly, we did not impose any exclusion criteria relating to a minimum or maximum amount of time between diagnosis and presentation or ART initiation for the control group. Baseline characteristics were compared between the study and control groups to assess equivalence.

Treatment adherence and retention in care were assessed in the study group only. Treatment adherence was measured as proportion of days covered based on pharmacy dispense data [20]. Retention in care was measured as the number of patients still on study at 48 weeks.

### Statistical analysis

Statistical analysis was completed using R programming [21]. All continuous variables were analyzed using Kruskal-Wallis or Wilcox rank test. The Fisher exact test was used to analyze categorical variables. A minimum study population size of 26 participants was deemed sufficient for a single-center pilot study, based on the number of treatment naïve PLWH who presented to Trillium in the preceding years; powered at 0.8 [22]. The population size was later expanded to 45 as a reflection of the increase in new diagnoses being seen at Trillium. The historical control population size was made to be similar to the study size by reviewing how many newly diagnosed patients presented each month prior to RSA initiation at Trillium and selecting the date range that met our population size needs.

### Care team

A multidisciplinary team of providers, care managers, pharmacists, STI testing and prevention specialists, and clinical laboratory services collaborated to create a consistent approach to RSA for all patients. Initial rapid point-of-care HIV testing was performed by Trillium’s STI testing specialists. These testing specialists complete thorough training and annual formal observations to ensure they are proficient at performing point-of-care HIV testing and are prepared to deliver results to patients.

The standard of care at Trillium includes care managers performing a comprehensive needs assessment during a new patient’s first visit to identify and address barriers to care and adherence. Care managers also connect patients with internal and community supportive services to address identified barriers. Internal supportive services available at Trillium include insurance enrollment and navigation, transportation assistance, food pantry access, housing support, and medication-assisted treatment (MAT). Community resources include legal aid services, employment services, and domestic violence programs.

An in-house pharmacy at Trillium provides same-day pick up, couriered, and mail delivered prescription medications. The pharmacists also monitor records of patients taking ART to ensure they have valid prescriptions for refills and have obtained their medications, providing another layer of adherence monitoring and support.

Having a clinical laboratory service which includes a phlebotomy center adjacent to Trillium’s main clinic allows convenient access for patients.

### Clinical and laboratory evaluations

Participants underwent clinical and laboratory evaluations at the time of enrollment and at subsequent follow-up visits according to the protocol in Appendix A, utilizing commercially available and validated assays. The rapid point-of-care HIV tests used are fourth generation (Determine™ HIV–1/2 Ag/Ab Combo immunoassay, Alere Inc.). The laboratory completes HIV-1 viral load quantification (COBAS 8800 system, Roche Diagnostics) and combined drug resistance and genotyping (GenoSure PRIme assay, Monogram Biosciences).

### Study oversight

The study protocol was reviewed and approved by an external accredited Institutional Review Board (IRB); WCG IRB (Puyallup, WA). Trillium contracted with a site management organization to manage study operations, maintain appropriate documentation, and report adverse events. All participants provided written informed consent and received compensation for their participation in the form of gift cards to local supermarkets.

### Screen failures

Participants had to complete at least one on-treatment visit and blood draw for viral load quantification to be included in statistical analysis. Some participants with false positive reactive tests (reactive rapid point-of-care HIV test with subsequent negative confirmatory testing) were noted. Those with negative confirmatory tests were instructed to stop taking ART and were excluded from the study. All efforts were made to retain these patients in care at Trillium and initiate HIV PrEP when appropriate.

## Results

### Baseline presentation and characteristics

**Fig. 1:**
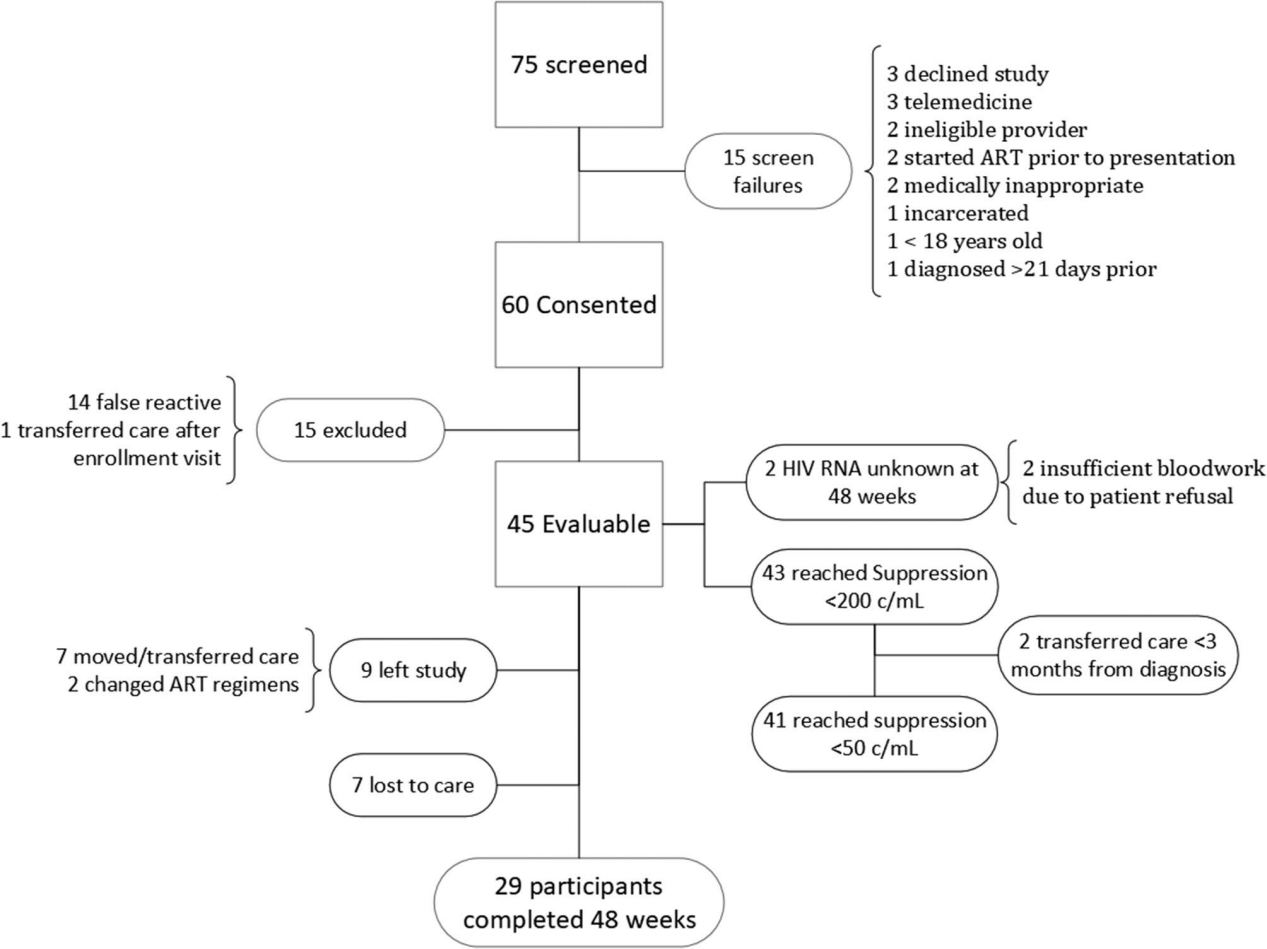
Screening, enrollment, and retention flowchart

From October 2018 to September 2021, 75 individuals presented to Trillium with a new or preliminary HIV diagnosis and 60 individuals consented to be in the study. All 60 participants agreed to RSA with B/F/TAF during their first HIV appointment with a provider. Fifteen participants were excluded due to false reactive point-of-care tests (14) or transfer of care immediately after diagnosis (1), resulting in study size of 45 participants (Fig. 1).

Twenty-six participants received their diagnosis at Trillium, either during routine screening through primary care (10), STI testing or treatment (8), screening for PrEP (7), or an at-home HIV test provided through Trillium’s remote testing service (1). Nineteen participants were diagnosed offsite through STI screening at an independent clinic or primary care office (7), an emergency department visit or hospital admission (5), the local Department of Health STI Clinic (4), a local university’s health center (2), or an over the counter at-home HIV test (1).

There were no statistically significant differences in baseline characteristics between the study RSA and historical control groups (Table 1).

**Table 1:** Baseline characteristics of study RSA and historical non-RSA control

Baseline characteristics	Study RSA (*n* = 45)	Non-RSA control (*n* = 42)	*P* value
Age at diagnosis, mean (SD)	31.7 (9.4)	37.7 (14.0)	0.093
Sex at birth = male, n (%)	43 (95.6%)	36 (85.7%)	0.148
Gender identity = male, n (%)	35 (77.8%)	33 (78.6%)	1.000
Sexual orientation = gay, n (%)	26 (57.8%)	20 (47.6%)	0.394
HIV viral load prior to ART initiation, median (IQR), log_10_ copies/mL	4.4 (3.8–5.1)	4.5 (4.1–5.1)	0.865
HIV viral load prior to ART initiation, median (IQR), copies/mL	49,158 (5,949 − 140,000)	31,500 (13,925 − 118,250)	0.865
Patients with HIV viral load prior to ART initiation ≥ 100,000 copies/mL, n (%)	13 (28.9%)	12 (28.6%)	1.000
Race, n (%)			
Race = white Race = black Race = other	25 (55.6%) 17 (37.8%) 1 (2.2%)	20 (47.6%) 18 (42.9%) 4 (9.5%)	0.523 0.667 0.154
Ethnicity, n (%)			
Ethnicity = Non-Hispanic/Latinx Ethnicity = Hispanic/Latinx Ethnicity = unreported	31 (68.9%) 11 (24.4%) 3 (6.7%)	36 (85.7%) 6 (14.3%) 0 (0.0%)	0.077 0.285 0.242
CD4 cells/mm^3^ prior to ART initiation, median (IQR)	458 (285–652)	381 (289–552)	0.290
CD4 < 200 cells/mm^3^ prior to ART initiation, n (%)	3 (6.7%)	5 (11.9%)	0.475

### False reactive tests

Fourteen of the 60 (23.3%) consented participants who had reactive point-of-care tests were determined to be HIV-negative upon confirmatory testing. All fourteen participants discontinued B/F/TAF, and three (21.4%) patients initiated oral PrEP within seven days of receiving their confirmatory negative HIV-1 RNA test results. No adverse events were reported while these patients were on B/F/TAF in absence of an HIV infection.

### Treatment initiation

The median number of days between HIV diagnosis and clinic presentation in the RSA group was significantly lower than that of the historical control group (Table 2). This same truncation was seen in time from clinic presentation to ART initiation. Commensurate with the preceding, time from diagnosis to ART initiations was significantly shorter in the study RSA group than in the non-RSA control group. In the study RSA group, 33 (76%) participants initiated ART within 3 days of their diagnosis.

**Table 2:** Clinic presentation and ART initiation of study RSA and historical non-RSA control

Outcomes	Study RSA *n* = 45	Non-RSA Control *n* = 42	*P* value
Diagnosis to clinic presentation, median (IQR)	0.0 (0.0–3.0) days	10.0 (5.0–34.75) days	< 0.001
Clinic presentation to ART, median (IQR)	0.0 (0.0–0.0) days	42.0 (28.25–64.75) days	< 0.001
Diagnosis to ART initiation, median (IQR)	0.0 (0.0–3.0) days	53.0 (42.25–95.25) days	< 0.001

All study RSA participants were on B/F/TAF for the entirety of their time on study. The non-RSA historical control patients were on a variety of regimens, with the largest proportion (45.2%) being on a single tablet regimen of elvitegravir, cobicistat, emtricitabine, and tenofovir (TDF or TAF; Appendix B).

### Virologic outcomes

There were no virologic failures noted through 48 weeks for those on B/F/TAF. Genotype testing was successfully completed for 39 (86.6%) participants during their time on study. The median time from diagnosis to receiving genotype laboratory results was 23 days (IQR 20–31 days). Among the 39 patients, transmitted drug resistance mutations were present in 10 (25.6%), 8 (20.5%) of which included a major NNRTI mutation. No participants had transmitted INSTI resistance. One participant had genotype results showing a resistance mutation (M184V) against emtricitabine after reaching viral suppression on B/F/TAF. No participants switched regimens due to genotype results. Two participants voluntarily changed regimens after reaching viral suppression, one for gastrointestinal side effects and the other because of a preference for a long-acting injectable.

Forty-three (95.6%) study participants had a documented viral load of < 200 copies/mL by week 48. The remaining 2 participants had an unknown viral load because of patient refusal of laboratory tests. Forty-one (91.1%) reached < 50 copies/mL while on study; the additional 2 participants who did not reach < 50 copies/mL ended study participation less than 3 months after diagnosis.

We compared the viral suppression outcomes of a historical non-RSA population (*n* = 42) to those of our study population. We found the durations from HIV diagnosis to viral suppression < 200 copies/mL and < 50 copies/mL were shorter in RSA patients than in non-RSA patients (*p* < 0.001; Table 3).

Furthermore, the times from ART initiation to viral suppression to < 200 copies/mL and to < 50 copies/mL were shorter in RSA patients than in non-RSA patients (*p* < 0.001). In the study group, 36 (80.0%) participants achieved virologic suppression in 30 days or less from ART initiation. All participants who achieved virologic suppression while on study did so in less than 6 months (maximum 163 days) from ART initiation.

**Table 3:** Time to virologic suppression in study RSA and historical non-RSA control

Outcomes	Study RSA *n* = 43	Non-RSA Control *n* = 42	*P* value
Diagnosis to viral load < 200 copies/mL, median (IQR)	21.0 (11.0–31.0) days	112 (81.5–196.0) days	< 0.001
ART to viral load < 200 copies/mL, median (IQR)	16.0 (9.0–29.0) days	34.5 (30.25–73.50) days	< 0.001
Diagnosis to viral load < 50 copies/mL, median (IQR)	32.0 (19.0–56.0) days*	181.0 (110.5–279.8) days	< 0.001
ART to viral load < 50 copies/mL, median (IQR)	28.0 (13.0–56.0) days*	62.0 (34.0–173.5) days	< 0.001

*n = 41

### Retention in care and treatment adherence

Twenty-nine (64.4%) participants were still engaged in study at 48 weeks. Nine participants left the study before 48 weeks because they transferred care (7) or changed regimens (2). Seven (15.5%) participants were considered lost to care at 48 weeks because they did not present for the final study visit. The median treatment adherence was 93.4% (interquartile range 76.5 − 99.4%), based on pharmacy dispense data.

### Patient acceptance of RSA

All enrolled participants started ART the same day as their first appointment. Twenty-seven participants completed the end of study questionnaire. Participant responses regarding how they felt starting ART immediately after their diagnosis were reviewed and several common themes were identified. The first and most common theme was general positivity, defined by feeling happy, good, supported, or relieved at starting ART. The second theme was an expressed readiness to start or not wanting to “waste time” before starting medication. The third theme was a sense of responsibility, which was often defined by the participant feeling ART initiation was the right thing to do for their health. Lastly, some participants expressed being overwhelmed by the diagnosis, but trusted the provider’s advice to start medication.

When asked how we could improve the rapid start process, the majority (21) of participants had no suggestions. Three participants suggested we have fewer team members at the first appointment.

### Real-world considerations

Of the 27 participants who completed the end of study questionnaire, 17 (62.9%) identified at least one barrier to care they experienced over the course of the study. The median number of barriers identified was 2 per participant. The most common barrier to care was lack of stable transportation (Table 4).

**Table 4:** Barriers to care in study RSA participants

Barrier to Care Identified	Study RSA Completed Questionnaire, *n* = 27
Unreliable source of transportation, n (%)	11 (40.7%)
Unstable housing, n (%)	10 (37.0%)
Food insecurity, n (%)	7 (25.9%)
Lack of insurance or underinsurance, n (%)	6 (22.2%)
Behavioral health concerns, n (%)	5 (18.5%)
Substance use, n (%)	5 (18.5%)

In March 2020, the COVID-19 pandemic significantly changed operations at the clinical site. The study protocol was amended to allow for virtual visits and delayed laboratory results. Study appointments transitioned from clinic visits to a majority telemedicine appointments, and participants without phones were given a phone through our Care Management program to complete their appointments. Participants were initially encouraged to delay completing bloodwork due to risk of exposure to COVID-19, but were able to complete STI testing via at home test kits that included self-swabs and a prepaid return envelope. Trillium provided free pharmacy delivery to the patients’ homes or location of their choosing. Patients who were homeless or unstably housed could pick up their medication “curbside,” with no direct contact.

## Discussion

This study shows that RSA is an effective model of care in a community health center setting and supports its role in ending the HIV epidemic. A daily single tablet regimen of B/F/TAF was effective for 95.6% participants to reach undetectable viral load in less than 6 months of ART initiation, despite variation in regimen adherence. Consistent with the literature [3–12], our study demonstrates that RSA shortens the amount of time from an initial HIV diagnosis to the ART initiation, consequently shortening the time from diagnosis to viral suppression. More rapid and sustained virologic suppression decreases opportunity for transmission. Some previous studies have not seen significant changes in time from ART initiation to viral suppression [8, 11] however, our study did show a shorter time from ART initiation to viral suppression.

The success of any treatment model depends on patients’ acceptance. Participants in this study were very agreeable to RSA. All study participants offered ART at their first visit accepted it. The standard questionnaire given at study completion showed participants were comfortable with the RSA process. Demonstrating patient acceptance in the diverse population from a community health center is important to getting other community providers to feel comfortable offering RSA to their patients.

Over the course of the study, we noted fourteen false reactive rapid point-of-care tests. Further analysis showed that these false reactive tests account for less than 0.2% of the tests performed at Trillium during the study time frame, which is within the published specificity rate [23]. Since the testing specialists are most often the patients’ first point of contact, it is important they are proficient in their testing role and promote confidence in the care team.

As a multidisciplinary community health center, Trillium was well positioned to take on this pilot study given its 30-year legacy in HIV prevention and treatment, and long-standing culture of early HIV intervention. Experience combined with evaluation of our programs supports our ability to navigate changing healthcare landscapes. In the wake of the COVID-19 crisis, Trillium had to create and implement new ways to care for PLWH. Though challenging, care was minimally interrupted, and study participants who were virally suppressed before the pandemic remained suppressed throughout. However, in various clinical settings, implementation and maintenance of RSA can present challenges. Provider availability can make it difficult to accommodate a new patient appointment on the same day. On-demand insurance navigation and enrollment services are necessary to provide HIV drug treatment at little to no cost for patients, which may not be available in all states. RSA requires the harmonized collaboration of service providers from across the care continuum, regardless of clinic size.

Our study has several limitations. The study was designed before RSA was included and defined as initiating ART within 72 hours of diagnosis in the New York State clinical guidelines [24]. Though our definition of RSA differs from current guidelines, the median and interquartile range of time from diagnosis to ART initiation in the study population mirrors the 72-hour window (Table 2). The majority (33, 76%) of participants met the 72-hour definition of RSA, and an additional 8 participants initiated therapy between 3 and 7 days from diagnosis. Although the study population was controlled for ART regimen variations by using only a single tablet regimen B/F/TAF, the historical control groups did not include this regimen because it was not yet approved by the FDA. For this reason, we are unable to identify whether the improved outcomes were more related to our RSA model versus the use of B/F/TAF. We are also unable to identify the specific elements of our RSA approach that influenced patients’ experiences the most. Use of a historical control group can create confounding issues, such as inadvertent omissions relating to retrospective data collection. To ensure the fairest comparison between the historical control and study group, we established baseline equivalence and mirrored our study screening method by including all the newly diagnosed patients from the established timeframe. Including all patients best captured the diversity in prior care models and avoided the influence of selection bias.

As a pilot study, our study population size was limited. Future studies should include larger populations with longer follow-up periods. The measure of treatment adherence used reflects the most adherent a patient could be given the amount of medication they have, which may not reflect the actual patient adherence. We attempted but were unable to complete accurate pill counts or obtain accurate reports of doses missed because participants frequently forgot to bring remaining medication with them to appointments.

## Conclusion

RSA is the emerging standard of care for HIV treatment globally [24, 25]. Our study shows that RSA can be successfully implemented in a real-world community health center. RSA shortens the time to undetectable viral load and fosters engagement in HIV care. The single tablet regimen of B/F/TAF for RSA is well accepted by patients and effective at suppressing HIV-1 viral load to undetectable levels. Efforts to end the HIV epidemic leverage treatment as prevention, and RSA expedites the process by getting PLWH on ART and virologically suppressed faster. More rapid virologic suppression impacts viral transmission at both the individual and community levels. RSA is an important tool for ending the HIV epidemic and real-world data from our study supports implementation in community health centers.

### Electronic supplementary material

Below is the link to the electronic supplementary material.

Supplementary material 1

## Data Availability

The datasets generated and analyzed during this study are not publicly available due to patient privacy and the sensitive nature of data related to HIV status. De-identified or limited data sets are available from the corresponding author (shilliard@trilliumhealth.org) on reasonable request.

## Abbreviations

RSA: Rapid start of antiretroviral therapy
ART: Antiretroviral therapy
B/F/TAF: Bictegravir, emtricitabine, and tenofovir alafenamide
TAF: Tenofovir alafenamide
TDF: Tenofovir disoproxil fumarate
Trillium: Trillium Health
FQHC: Federally qualified health center
PLWH: People living with HIV
HIV: Human immunodeficiency virus
HIV-1: Human immunodeficiency virus type 1
MAT: Medication-assisted treatment
IRB: Institutional Review Board
PrEP: Pre-exposure prophylaxis
STI: Sexually Transmitted infections
RNA: Ribonucleic Acid
NNRTI: Non-nucleoside reverse transcriptase inhibitors
INSTI: Integrase strand transfer inhibitors
FDA: U.S. Food and Drug Administration
IQR: Interquartile range
