# Supplementary material for: Real world community-based HIV Rapid Start Antiretroviral with B/F/TAF versus prior models of antiretroviral therapy start – the RoCHaCHa study, a pilot study
Source: AIDS Res Ther. 2024 Jul 10;21:45. doi: 10.1186/s12981-024-00631-6 (PMC11238360; doi:10.1186/s12981-024-00631-6)
Supplement: Supplementary file 1 — Supplementary material 1 [file 12981_2024_631_MOESM1_ESM.docx]

Appendix A

| **RSA Procedure and Follow-up Schedule** | | **Trillium Health Rapid Start Antiretroviral Pilot** | | | | |  |  |  |
| --- | --- | --- | --- | --- | --- | --- | --- | --- | --- |
| **Procedure** | **Day 1** | **Day 7 +/- 3 days** | **Day 14 +/- 3 days** | **Day 28 +/- 5 days** | **Day 56 +/- 7 days** | **Day 84 +/- 14 days** | **Day 128 +/- 14 days** | **Day 252 +/- 14 days** | **Day 336 +/- 14 days** |
| Inclusion/Exclusion criteria | X |  |  |  |  |  |  |  |  |
| Informed Consent | X |  |  |  |  |  |  |  |  |
| Medical History | X |  |  |  |  |  |  |  |  |
| Concomitant medications | X | X | X | X | X | X | X | X | X |
| Adherence Check |  | X | X | X | X | X | X | X | X |
| Adverse Events | X | X | X | X | X | X | X | X | X |
| Complete/symptom-directed Physical exam | X |  |  | X | X | X | X | X | X |
| 12 lead ECG | X |  |  |  |  |  |  |  |  |
| Height | X |  |  |  |  |  |  |  |  |
| VS and Weight | X |  |  | X | X | X | X | X | X |
| Urinalysis | X |  |  | X | X | X | X | X | X |
| Serum Pregnancy * | X |  |  |  |  |  |  |  |  |
| Urine Pregnancy test* |  |  |  | X | X | X | X | X | X |
| Chemistry profile | X |  |  | X | X | X | X | X | X |
| eGFR | X |  |  | X | X | X | X | X | X |
| Hematology profile | X |  |  | X | X | X | X | X | X |
| Metabolic assessment^ | < |  |  |  |  | X | X |  | X |
| Plasma HIV-1 RNA | X | X | X | X | X | X | X | X | X |
| CD4+ Cell Count and percentage | X | X | X | X | X | X | X | X | X |
| Hepatitis Serology. HCV RNA if clinically needed. | X |  |  |  |  |  |  |  | X |
| Plasma HBV DNA~ | X |  |  | X | X | X | X | X | X |
| HIV-1 Genotype and Phenotype | X |  |  |  |  |  |  |  |  |
| STI Screeningª | X |  |  |  |  | X | X | X | X |
| Care Managementª | X |  |  |  |  |  |  |  |  |

*Subjects of child bearing potential

^Fasting lipid test

ª additional follow up as needed

~ if HBV positive

< Within 21 days because it is unlikely that the subject will be fasting on day 1.

Appendix B

ART Regimens of Historical Non-RSA Control

| Regimen | Portion of non-RSA control (n=42),  n (%) |
| --- | --- |
| ABC/3TC +DTG | 1 (2.4%) |
| DRV/COBI + TAF/FTC | 1 (2.4%) |
| EVG/COBI/TAF/FTC + DRV | 1 (2.4%) |
| FTC/TDF + RAL | 1 (2.4%) |
| DRV/COBI + TDF/FTC | 2 (4.8%) |
| DTG + TAF/FTC | 2 (4.8%) |
| TDF/FTC + RAL or MK-0518  (MK-0158-929 trial) | 2 (4.8%) |
| DRV/COBI + DTG | 3 (7.1%) |
| DTG + TDF/FTC | 3 (7.1%) |
| DTG/ABC/3TC | 7 (16.6%) |
| EVG/COBI/TDF/FTC | 9 (21.4%) |
| EVG/COBI/TAF/FTC | 10 (23.8%) |
